# Mammary secretory carcinoma in a female domestic rabbit (Oryctolagus cuniculus): histopathological and immunohistochemical features

**DOI:** 10.1007/s11259-026-11513-9

**Published:** 2026-10-06

**Authors:** Maíra Meira Nunes, Eric Santos Oliveira, Júlia Gabriela Wronski, Rayane de Souza Ruas Lopes, Marcella Letícia Melo Souza da Rocha, Karen Yumi Ribeiro Nakagaki, Geovanni Dantas Cassali

**Affiliations:** Laboratory of Comparative Pathology, Institute of Biological Sciences, Federal University of Minas Gerais, Belo Horizonte, Minas Gerais Brazil

**Keywords:** Oryctolagus cuniculus, Mammary neoplasm, Immunohistochemistry, α-lactalbumin, Secretory carcinoma

## Abstract

Secretory carcinoma of the breast is a rare histological subtype in women and is uncommon in female dogs and cats, with only two cases described in female dogs and one case described in a cat. This report describes the histopathological and immunohistochemical features of secretory carcinoma of the mammary gland in a domestic rabbit (*Oryctolagus cuniculus*). Microscopic evaluation revealed a non-encapsulated, well-demarcated neoplastic proliferation composed of epithelial cells arranged in solid and tubular patterns, supported by a delicate fibrous connective tissue stroma. The neoplastic cells had slightly eosinophilic cytoplasm, frequently containing large, well-demarcated vacuoles that displaced the nucleus to the periphery, giving the cells a signet-ring-like appearance. Periodic acid-Schiff (PAS) histochemical staining, performed with and without diastase digestion, showed positivity in the intracellular and extracellular secretory material in both approaches. Immunohistochemical evaluation was performed for the markers α-lactalbumin, Ki-67, estrogen receptor, progesterone receptor, and COX-2. Positive cytoplasmic immunolabeling for α-lactalbumin was observed in neoplastic cells and in intracellular and extracellular secretory material, confirming their secretory nature. The diagnosis of secretory carcinoma was established based on the association of histopathological, histochemical, and immunohistochemical features. Considering the secretory features of the mammary gland in rabbits, secretory carcinoma should be included in the differential diagnosis of mammary carcinomas with prominent secretory activity in this species.

## Introduction

Mammary neoplasms are frequently described in domestic rabbits (*Oryctolagus cuniculus*), and carcinomas predominate among the mammary proliferative lesions reported in this species (Baum and Hewicker-Trautwein 2015; Schöniger et al. 2019). Retrospective studies in domestic rabbits document a significant number of mammary tumors in routine diagnostic practice, reinforcing the clinical relevance of these neoplasms (Schöniger et al. 2014; Bertram et al. 2021). Despite the growing recognition of mammary neoplasms in rabbits and the proposal of a species-specific histological classification for mammary tumors by Baum and Hewicker-Trautwein (2015), an internationally standardized classification system is still lacking, mainly owing to the scarcity of systematic studies in this species and the limited knowledge of prognostic factors in these animals (Schöniger et al. 2019). In contrast, for female dogs and cats, well-founded classification systems and well-established, internationally accepted consensus statements are available (Goldschmidt et al. 2011; Cassali et al. 2014, 2020; Zappulli et al. 2019; Cassali et al. 2024).

Secretory carcinoma of the breast is an extremely rare histological subtype, recognized by the World Health Organization classification of human breast tumors, accounting for less than 0.15% of infiltrative breast carcinomas in women (Vasudev and Onuma 2011; Krings et al. 2019; Min et al. 2022). It was first described by McDivitt and Stewart in 1966 as juvenile carcinoma, because the first cases occurred in children and young women. Later, after similar tumors were described in adult patients, this designation was replaced by the term secretory carcinoma (Tavassoli and Norris 1980).

In veterinary medicine, secretory carcinoma was first described in the mammary gland of a female dog by Cassali et al. (1999) in *Veterinary Pathology*, based on its histological and immunohistochemical similarity to human secretory carcinoma. It was subsequently incorporated as a distinct histological subtype in the classifications proposed by Cassali et al. (2014, 2020). Terminological differences exist among veterinary mammary tumor classification systems, and in some classifications the term “secretory” has been used in association with lipid-rich carcinoma (Goldschmidt et al. 2011; Zappulli et al. 2019). In contrast, the classifications proposed by Cassali et al. (2014, 2020) consider secretory carcinoma and lipid-rich carcinoma as distinct histological entities, based on their different histomorphological, histochemical, and immunohistochemical features. In rabbits, secretory carcinoma is not included as a distinct subtype in the mammary tumor classification proposed by Baum and Hewicker-Trautwein (2015).

To date, only two cases of secretory mammary carcinoma have been described in the veterinary literature in female dogs (Cassali et al. 1999; Silva et al. 2022) and one in situ case in a cat (Dos Reys et al. 2025). The small number of reported cases in animals means that epidemiological and clinicopathological data remain quite scarce. However, terminological and classificatory differences may also lead to neoplasms with secretory features being classified under other histological subtypes. To date, there are no reports of mammary secretory carcinoma in domestic rabbits.

Histologically, secretory carcinoma is characterized by proliferation of epithelial cells arranged predominantly in solid and tubular patterns, associated with the presence of intracellular and extracellular secretory material. The neoplastic cells have abundant vacuolated cytoplasm, often with peripheral nuclear displacement and a signet-ring-like appearance (Vasudev and Onuma 2011; Eusebi et al. 2012; Cassali et al. 2014; Cassali and Nakagaki 2023). Immunohistochemical evaluation for α-lactalbumin aids diagnosis by demonstrating secretory differentiation in the neoplastic cells and in the intra- and extracellular secretory material, and its expression should be interpreted together with the histopathological findings (Vasudev and Onuma 2011; Eusebi et al. 2012; Min et al. 2022; Cassali and Nakagaki 2023).

The aim of this study is to report a case of secretory carcinoma of the mammary gland in a female rabbit and to describe its histomorphological, histochemical, and immunohistochemical features.

## Case presentation

A female domestic rabbit (*Oryctolagus cuniculus*), of unreported age, presented with a nodule located in the left thoracic mammary gland, measuring 1.9 × 1.3 × 0.5 cm, not adherent to the underlying musculature, non-ulcerated, and with an irregular surface. The patient also had a nodule located between the right caudal abdominal and inguinal mammary glands, measuring 1.1 × 0.7 × 0.3 cm, not adherent to the underlying musculature, non-ulcerated, and with a smooth surface, as well as a mass in the left uterine horn, measuring 4.6 × 3.7 × 4.8 cm. Surgical excision of the mammary nodules and ovariohysterectomy were performed. The tissues were fixed in 10% neutral buffered formalin, submitted for routine histological processing, and subsequently stained with hematoxylin and eosin (HE).

Macroscopically, the nodule located in the thoracic mammary gland had a firm consistency, solid surface, homogeneous appearance, and brown coloration. On cut surface, the nodule located between the caudal abdominal and inguinal mammary glands showed cystic cavities, the largest measuring 0.3 × 0.3 cm, filled with light-brown viscous material. On cut surface, the uterine horn nodule had a solid surface, heterogeneous appearance, light-brown to brown coloration, and multiple cystic cavities, the largest measuring 0.5 × 0.4 cm, containing translucent fluid.

**Fig. 1:**
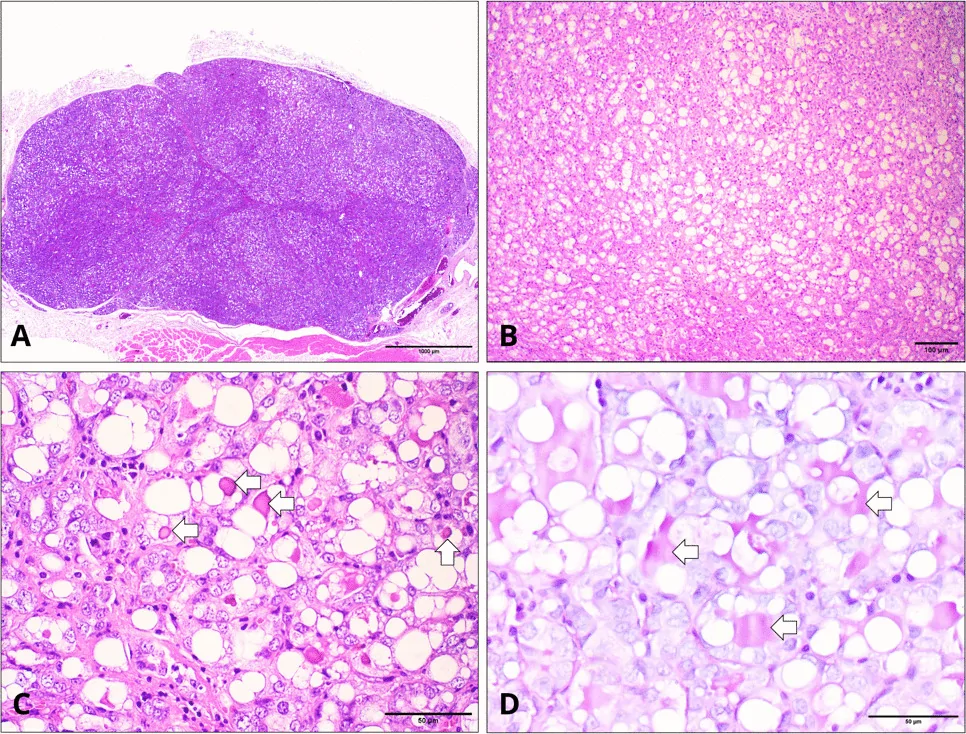
Mammary secretory carcinoma in a rabbit. **A** Well-demarcated neoplastic proliferation. (HE; 2x). **B** Neoplastic proliferation in solid and tubular patterns. (HE; 10x). **C** Neoplastic cells arranged in tubules, frequently containing large, well-demarcated vacuoles that displaced the nucleus to the periphery, giving a signet-ring appearance. Amorphous, eosinophilic, homogeneous material is observed within the lumen of the tubules and in the cytoplasm of some neoplastic cells. (HE; 40x). **D** PAS-positive secretory material, without diastase digestion. (PAS; 40x)

Histologically, a non-encapsulated, well-demarcated neoplastic proliferation was observed affecting the thoracic mammary gland (Fig. 1A), with neoplastic cells arranged in solid and tubular patterns, delimited by a delicate fibrous connective tissue stroma (Fig. 1B). The cells had slightly eosinophilic cytoplasm, frequently containing large, well-demarcated vacuoles that displaced the nucleus to the periphery, giving them a signet-ring-like appearance. The nuclei were round to oval, with loose chromatin and distinct, occasionally multiple, nucleoli. Amorphous, eosinophilic, homogeneous material was observed within the lumen of the tubules (Fig. 1C). Moderate anisocytosis and anisokaryosis, a moderate nuclear-to-cytoplasmic ratio, rare cells exhibiting karyomegaly, and 23 mitotic figures in 10 high-power fields (400x/2.37 mm^2^) were observed. Areas of moderate fibrosis were also noted. In the adjacent mammary gland, mild ductal ectasia was observed, with ducts containing homogeneous eosinophilic secretion. Based on the histopathological findings, lipid-rich carcinoma and secretory mammary carcinoma were considered the main diagnostic hypotheses, owing to the presence of vacuolated cells and amorphous eosinophilic intracellular and extracellular material, according to the classification proposed by Cassali et al. (2014) for canine mammary neoplasms.

Periodic acid-Schiff (PAS) histochemical staining, with and without diastase digestion, was performed using reagents from Apta Biotech (Brazil), according to the manufacturer's protocol. In both approaches, positivity was observed in the intracellular and extracellular secretory material (Fig. 1D).

Given the diagnostic hypotheses considered, immunohistochemical evaluation was used to establish the diagnosis and better characterize the neoplasm. Immunohistochemistry was performed on 4 μm histological sections obtained from paraffin-embedded blocks and mounted on slides previously treated with gelatin. The antibody panel included α-lactalbumin, COX-2, Ki-67, estrogen receptor (ER), and progesterone receptor (PR). Antigen retrieval was performed in wet heat at 125 °C, except for α-lactalbumin, for which retrieval was performed at 95 °C. Sections were incubated with the primary antibodies for 16 h at 4 °C. Detection was performed using the streptavidin–biotin-peroxidase method, according to the manufacturer's recommendations, and reactions were visualized by incubating the slides with the chromogen 3,3'-diaminobenzidine tetrahydrochloride (DAB Substrate System, Dako, Carpinteria, CA, USA), followed by Mayer's hematoxylin counterstaining. Normal rabbit mammary gland was used as the positive control. For the negative control, the primary antibody was replaced with buffer solution, while the remaining steps of the immunohistochemical protocol were kept unchanged. Antibody specifications and evaluation criteria are detailed in Table 1.

**Table 1:** Antibody specifications and evaluation criteria of mammary secretory carcinoma in a domestic rabbit (*Oryctolagus cuniculus*)

Antibody	Clone	Manufacturer	Dilution	Evaluation criteria
α-lactalbumin	Polyclonal	Dako	1:500	Cytoplasmic immunolabeling in neoplastic cells and secretory material (Cassali et al. 1999; Vasudev and Onuma 2011; Eusebi et al. 2012)
COX-2	SP21	Invitrogen	1:50	Cytoplasmic immunolabeling evaluated according to the percentage of positive cells, on a scale of 0 to 4, and labeling intensity, on a scale of 0 to 3. The final score was obtained by multiplying these values (Lavalle et al. 2009)
Ki-67	MIB-1	Dako	Ready-to-use	Presence of nuclear immunolabeling. The proliferative index was determined by the percentage of positive nuclei among 500 neoplastic cells evaluated in the areas of highest immunolabeling (Dutra et al. 2008)
Estrogen receptor (ER)	ID5	Dako	Ready-to-use	Nuclear immunolabeling in more than 1% of neoplastic cells, based on the percentage of positive cells (Hammond et al. 2010)
Progesterone receptor (PR)	HPRA-2	Invitrogen	1:50	Nuclear immunolabeling in more than 1% of neoplastic cells, based on the percentage of positive cells (Hammond et al. 2010)

Immunolabeling was evaluated qualitatively for α-lactalbumin, quantitatively for Ki-67, and semiquantitatively for COX-2, ER, and PR.

**Fig. 2:**
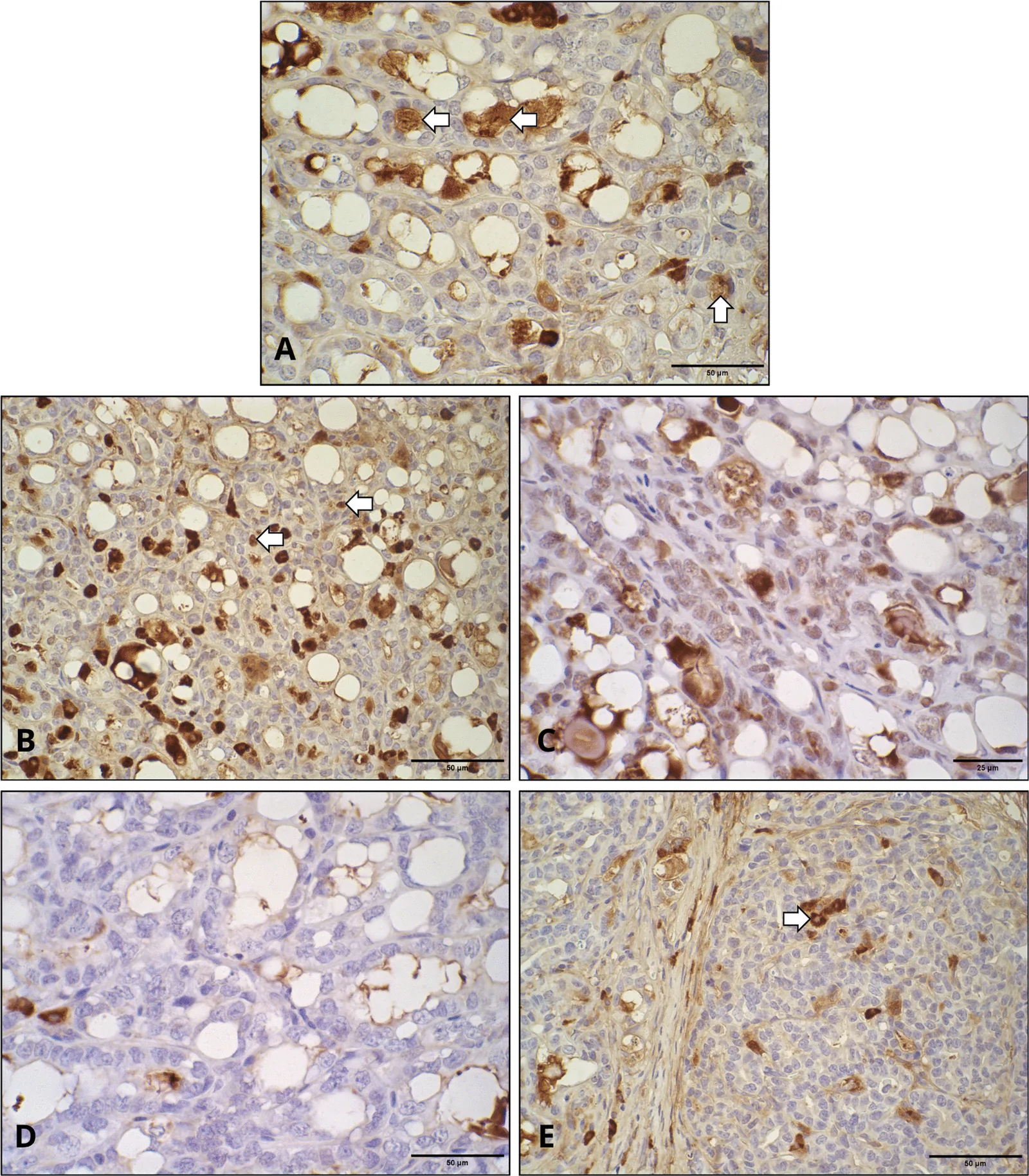
Immunohistochemistry of mammary secretory carcinoma in a rabbit. Positive immunoreactivity appears as brown staining. **A** α-lactalbumin: cytoplasmic immunostaining in neoplastic cells and luminal secretion (arrows) (40x). **B** Ki-67: nuclear immunostaining in some neoplastic cells (arrows) (40x). **C** PR: nuclear immunostaining in neoplastic cells (40x). **D** ER: absence of nuclear immunostaining in neoplastic cells (40x). (E) COX-2: strong cytoplasmic immunostaining (intensity score 3) in neoplastic cells (arrow) (40x)

The neoplastic cells showed positive cytoplasmic immunolabeling for α-lactalbumin, with labeling also observed in the secretory material (Fig. 2A). Nuclear immunolabeling for Ki-67 was positive in approximately 26% of neoplastic cells (Fig. 2B). The progesterone receptor showed positive nuclear immunolabeling in 51–75% of neoplastic cells (+ + +) (Fig. 2C), whereas no nuclear immunolabeling was observed for the estrogen receptor (Fig. 2D). For COX-2, strong cytoplasmic immunolabeling was observed in fewer than 10% of neoplastic cells (Fig. 2E).

Integration of the histomorphological, histochemical, and immunohistochemical findings confirmed the diagnosis of secretory carcinoma.

Histopathological evaluation of the nodule located between the caudal abdominal and inguinal mammary glands revealed granulomatous mastitis associated with fibrosis and cystic cavity formation, whereas the uterine horn mass was diagnosed as leiomyosarcoma. No histopathological changes were observed in the ovaries, and the lymph nodes were not submitted for analysis.

## Discussion and conclusions

In human medicine, secretory carcinoma is described as an invasive mammary neoplasm composed of epithelial cells with intracytoplasmic secretory vacuoles and extracellular eosinophilic secretion. Its histological architecture is variable and may include microcystic, solid, tubular, and papillary patterns, often combined within the same tumor (Eusebi et al. 2012; Krings et al. 2019). These morphological features are similar to those observed in the present case, in which the neoplasm showed solid and tubular patterns associated with intracellular and extracellular eosinophilic amorphous secretory material, as well as vacuolated cells frequently showing a signet-ring appearance. In veterinary medicine, the cases of mammary secretory carcinoma described to date in female dogs and in a cat are also similar to those reported in women. In the two canine cases, a solid growth pattern predominated (Cassali et al. 1999; Silva et al. 2022), whereas the case described in a cat showed an in situ tubular pattern (Dos Reys et al. 2025), which is also recognized among the histological presentations of this subtype in women (Krings et al. 2019). In the present report, a solid growth pattern was observed.

In women and female dogs, secretory carcinoma must be differentiated from other mammary neoplasms that may show similar morphological features. In human medicine, the main differential diagnoses include carcinoma with apocrine differentiation, acinic cell carcinoma, and cystic hypersecretory carcinoma (Osako et al. 2013; Krings et al. 2019). In female dogs, neoplasms containing intracellular and extracellular material, such as lipid-rich carcinoma, glycogen-rich carcinoma, and mucinous carcinoma, should be included among the differential diagnoses (Cassali et al. 2014; Cassali and Nakagaki 2023). The distinction among these subtypes is supported by evaluation of α-lactalbumin immunoreactivity and by the use of histochemical techniques such as PAS, with or without diastase digestion. In secretory carcinoma, the intracellular and extracellular content is PAS-positive, both before and after diastase digestion (Cassali et al. 1999; Vasudev and Onuma 2011; Eusebi et al. 2012; Min et al. 2022), a finding that corroborates the results observed in the present case, in which PAS positivity was also demonstrated before and after diastase digestion. In glycogen-rich carcinoma, the intracytoplasmic content is PAS-positive but loses staining after diastase digestion; in mucinous carcinoma, the extracellular content (mucin) is weakly PAS-positive and strongly Alcian blue-positive; by contrast, in lipid-rich carcinoma, neoplastic cells are PAS-negative (Cassali and Nakagaki 2023).

In the present case, positive immunolabeling for α-lactalbumin, observed in the cytoplasm of neoplastic cells and in intracellular and extracellular secretory content, supported the secretory differentiation of the neoplasm and, together with the histopathological and histochemical findings, corroborated the diagnosis of secretory carcinoma. α-lactalbumin is a protein related to milk secretion, and its expression has been described as a useful finding in the characterization of this tumor type (Vasudev and Onuma 2011; Osako et al. 2013; Min et al. 2022; Silva et al. 2022). In female dogs, Cassali et al. (1999) reported strong immunoreactivity for α-lactalbumin in the neoplastic cells of a mammary secretory carcinoma, reinforcing the morphological and immunophenotypic similarity between the cases described in veterinary and human medicine.

The histochemical findings supported the secretory nature of the neoplasm, as the intracellular and extracellular secretory material remained PAS-positive after diastase digestion. In addition, immunoreactivity for α-lactalbumin was observed in the neoplastic cells and luminal secretion, further supporting secretory differentiation. In human secretory breast carcinoma, additional histochemical methods, including Alcian blue, AB-PAS, and mucicarmine, may also be used to characterize the secretory material (Boustros et al. 2024). Although these techniques may provide complementary information, the association of the characteristic histomorphological features, PAS positivity retained after diastase digestion, and α-lactalbumin immunoreactivity supported the diagnosis in this case. Lipid-rich carcinoma represents one of the main differential diagnoses of secretory carcinoma. According to Cassali et al. (2014), secretory carcinoma and lipid-rich carcinoma represent distinct histological entities, with the latter characterized by intracytoplasmic lipid accumulation that can be demonstrated by Oil Red O or Sudan staining in frozen sections. Frozen tumor tissue was not available for lipid-specific staining in this case.

Secretory activity is frequently reported in mammary proliferative lesions and carcinomas of rabbits, characterized by the presence of eosinophilic material and neoplastic cells with clear, large cytoplasmic vacuoles (Schöniger et al. 2014; Degner et al. 2018). These findings may be associated with the secretory activity of the mammary gland in this species, given the high lipid and protein content of rabbit milk (Schöniger et al. 2019). In this case, the presence of intracellular and extracellular eosinophilic secretion, intracytoplasmic vacuolization, and α-lactalbumin immunoreactivity supported the diagnosis of secretory carcinoma. Thus, in mammary carcinomas of rabbits with prominent secretory activity, α-lactalbumin evaluation may be useful as a complementary tool for phenotypic characterization of the neoplasm and to assist in the differential diagnosis of other subtypes, such as lipid-rich carcinoma.

In secretory carcinoma of the breast in women, the Ki-67 index is generally low, with a mean value of approximately 10% (Li et al. 2019). In the present case, Ki-67 immunolabeling was observed in 26% of neoplastic cells, indicating high proliferative activity. Although this marker is widely used in the evaluation of mammary neoplasms in women (Nielsen et al. 2021), female dogs, and cats (Cassali et al. 2020), its application in mammary tumors of rabbits is not yet well established. In available studies on proliferative lesions and mammary tumors in this species, proliferative activity has been described mainly based on mitotic count, which may vary widely among mammary carcinomas, from 0 to 36 mitotic figures in 10 high-power fields (Baum and Hewicker-Trautwein 2015; Schöniger et al. 2014; Degner et al. 2018). In this case, 23 mitotic figures were observed in 2.37 mm^2^.

In canine mammary neoplasms, immunohistochemical evaluation of hormone receptors has been used to investigate possible clinicopathological and prognostic associations (Millanta et al. 2005; Peña et al. 2014). In rabbits, however, receptor expression in mammary tumors remains poorly studied. Degner et al. (2018) analyzed estrogen receptor (ER) and progesterone receptor (PR) expression in proliferative lesions of the mammary gland from 124 pet rabbits, of which 119 corresponded to mammary carcinomas. Most carcinomas were negative for both receptors (75/119). ER immunolabeling was observed in 20% (24/119) of carcinomas, PR immunolabeling in 35% (32/119), and coexpression of both receptors in 18% (22/119) of cases. In the present report, positive nuclear immunolabeling for PR was observed in 51–75% of neoplastic cells (+ + +), with absence of ER immunolabeling, a profile that is uncommon among the mammary carcinomas of rabbits described by Degner et al. (2018). In a study involving 228 canine mammary tumors, 98 showed an ER-negative, PR-positive immunophenotype (Martín de las Mulas et al. 2005). The authors suggested that isolated PR expression could reflect partial maintenance of a degree of hormonal influence on the tumor and raised the possibility of evaluating PR antagonists as adjuvant therapy after surgical excision. In rabbits, in which surgery remains the main therapeutic option for mammary masses (Schöniger et al. 2019), further studies are still needed to clarify the biological significance of this immunophenotype and to determine whether ER and PR expression may have prognostic value or therapeutic utility.

To date, COX-2 expression in mammary neoplasms of domestic rabbits has not been documented in the literature. COX-2 was included in the immunohistochemical panel to further characterize the biological behavior of the neoplasm, given its involvement in tumor progression and angiogenesis. In mammary carcinomas of female dogs, increased COX-2 expression has been associated with greater angiogenesis, worse prognosis, and shorter overall survival (Lavalle et al. 2009). In rabbits, although its expression has not yet been investigated in mammary neoplasms, COX-2 immunoreactivity has already been demonstrated in uterine adenocarcinomas, suggesting that this protein may be expressed by epithelial neoplasms in this species (Vaccaro et al. 2024). In the present case, strong cytoplasmic immunolabeling was observed in fewer than 10% of neoplastic cells, resulting in a score of 3, compatible with weak immunoreactivity according to the evaluation system used by Vaccaro et al. (2024). This finding shows that the secretory carcinoma had some degree of COX-2 expression and contributes to its immunophenotypic characterization. However, given the absence of specific data on mammary neoplasms in rabbits, studies with a larger number of cases are needed to determine the frequency of COX-2 expression and its possible association with clinicopathological features, biological behavior, prognosis, and response to inhibitors of this enzyme.

In rabbits, cystic mammary lesions may occur concomitantly with proliferative and neoplastic changes (Schöniger et al. 2019). In the study by Schöniger et al. (2014), multiple mammary cysts were observed in 10 of 24 pet rabbits, and 10 animals had more than one type of mammary lesion, indicating that different histological patterns may coexist in the mammary gland of this species. In this case, in addition to secretory carcinoma, mammary cysts and mastitis were identified in another mammary gland. Mastitis is described in rabbits, especially in lactating or pseudopregnant females, and may be related to local predisposing factors such as trauma, inadequate hygiene conditions, and bacterial infections (Hughes and Watson 2018). In addition to mammary lesions, a uterine neoplasm diagnosed as leiomyosarcoma was identified. In a retrospective analysis of 50 rabbits with uterine disorders, Künzel et al. (2015) reported a low frequency of this neoplasm, with uterine leiomyosarcoma identified in four cases, either alone or in association with other uterine changes, including adenocarcinoma, the main uterine neoplasm in rabbits, and endometrial hyperplasia. The concomitant occurrence of mammary and uterine neoplasms has already been described in rabbits (Schöniger et al. 2014; Baum and Hewicker-Trautwein 2015; Degner et al. 2018; Bertram et al. 2021). However, there is insufficient evidence to establish a direct hormonal or pathogenetic association between these neoplasms (Schöniger et al. 2019).

In women, secretory carcinoma of the breast is generally considered a low-grade neoplasm with favorable prognosis (Vasudev and Onuma 2011; Eusebi et al. 2012; Min et al. 2022). However, regional lymph node involvement and, less frequently, distant metastases have been reported (Zhao et al. 2024; Boustros et al. 2024). Recent morphologic and molecular findings suggest that a subset of tumors may exhibit more aggressive behavior, particularly those with a predominantly solid growth pattern, celular atypia increased, higher mitotic activity, and necrosis. Nevertheless, the prognostic significance of these features remains uncertain and requires further investigation (Ren et al. 2025). In veterinary medicine, however, there are few reports of this subtype, and in the cases described in female dogs, the occurrence of metastases in different organs and bone tissue suggests that secretory carcinoma may have a more aggressive biological behavior and a guarded prognosis (Cassali et al. 1999; Silva et al. 2022). In the case described here, information on treatment, postoperative follow-up, and survival of the rabbit was not available, precluding assessment of the clinical course and prognosis of this neoplasm in this species.

This is the first report of secretory carcinoma in the mammary gland of a domestic rabbit, diagnosed based on the association of histomorphological, histochemical, and immunohistochemical findings. The characterization of this histological subtype adds to the still limited number of spontaneous mammary neoplasms documented in domestic rabbits and provides data that may contribute to the development of a more comprehensive classification system for these tumors. Considering the secretory features of the mammary gland in this species, secretory carcinoma should be included among the differential diagnoses of mammary carcinomas with prominent secretory activity and similar architectural patterns. Given the rarity of mammary secretory carcinoma in female rabbits and the still-limited knowledge regarding this histological subtype in the species, further studies are needed to better characterize its biological behavior, prognosis, and clinical course, although the low incidence of such cases may limit the availability of larger case series.

## Data Availability

No datasets were generated or analysed during the current study.

## References

[CR1] Baum B, Hewicker-Trautwein M (2015) Classification and epidemiology of mammary tumours in pet rabbits (*Oryctolagus cuniculus)*. J Comp Pathol 152(4):291–298. 10.1016/j.jcpa.2015.01.009 25840882

[CR2] Bertram CA, Bertram B, Bartel A, Ewringmann A, Fragoso-Garcia MA, Erickson NA, Müller K, Klopfleisch R (2021) Neoplasia and tumor-like lesions in pet rabbits (*Oryctolagus cuniculus*): a retrospective analysis of cases between 1995 and 2019. Vet Pathol 58(5):901–911. 10.1177/0300985820973460 33213301

[CR3] Boustros P, Sanchez LM, Gaboury L, El Khoury M (2024) Secretory carcinoma of the breast: radiologic-pathologic correlation. J Breast Imaging 6(5):520–528. 10.1093/jbi/wbae041 39259928

[CR4] Cassali GD, Nakagaki KYR (2023) Patologia Mamária Canina e Felina – Do Diagnóstico ao Tratamento [Canine and Feline Mammary Pathology: From Diagnosis to Treatment; in Portuguese]. MedVet, São Paulo

[CR5] Cassali GD, Gobbi H, Gartner F, Schmitt FC (1999) Secretory carcinoma of the canine mammary gland. Vet Pathol 36(6):601–603. 10.1354/vp.36-6-601 10568441

[CR6] Cassali GD, Lavalle GE, De Nardi AB, Ferreira E, Bertagnolli AC, Estrela-Lima A, Alessi AC, Daleck CR, Salgado BS, Fernandes CG, Sobral RA, Amorim RL, Gamba CO, Damasceno KA, Auler PA, Magalhães GM, Silva JO, Raposo JB, Ferreira AMR, Oliveira LO, Malm C, Zuccari APC, Tanaka NM, Ribeiro LR, Campos LC, Souza CM, Leite JS, Soares LMC, Cavalcanti MF, Fonteles ZGC, Schuch ID, Paniago J, Oliveira TS, Terra EM, Castanheira TLL, Felix AOC, Carvalho GD, Guim TN, Guim TN, Garrido E, Fernandes SC, Maia FCL, Dagli MLZ, Rocha NS, Fukumasu H, Grandi F, Machado JP, Silva MMS, Bezerril JE, Frehse MS, Paes de Almeida EC, Campos CB (2014) Consensus for the diagnosis prognosis and treatment of canine mammary tumours. Braz J Vet Pathol 7(2):38–69

[CR7] Cassali GD, Jark PC, Gamba C, Damasceno KA, Lima AE, Nardi ABD, Ferreira E, Horta RS, Firmo BF, Sueiro FAR, Rodrigues LCS, Nakagaki KYR (2020) Consensus regarding the diagnosis, prognosis and treatment of canine and feline mammary tumors - 2019. Braz J Vet Pathol 13(3):555–574. 10.24070/bjvp.1983-0246.v13i3p555-574

[CR8] Cassali GD, Nakagaki KYR, Jark PC, Horta RS, Arias AC, Tellado MN, Estrela-Lima A, Sueiro FAR, Reys MP, Wronski JG, Sierra OR, Kristancic EA, Decuadro A, Gómez JLA (2024) Consensus on the diagnosis, prognosis, and treatment of canine and feline mammary tumors: solid arrangement – 2023. Braz J Vet Pathol 17(3):152–163. 10.24070/bjvp.1983-0246.v17i3p152-163

[CR9] Degner S, Schoon HA, Laik-Schandelmaier C, Aupperle-Lellbach H, Schöniger S (2018) Estrogen receptor-α and progesterone receptor expression in mammary proliferative lesions of female pet rabbits. Vet Pathol 55(6):838–848. 10.1177/0300985818788611 30114981

[CR10] Dos Reys MP, Souza FR, Viscone ÉA, Nakagaki KYR, Cassali GD (2025) Secretory mammary carcinoma in situ in a cat. Acta Sci Vet 53. 10.22456/1679-9216.142887

[CR11] Dutra AP, Azevedo Júnior GM, Schmitt FC, Cassali GD (2008) Assessment of cell proliferation and prognostic factors in canine mammary gland tumors. Arq Bras Med Vet Zootec 60(6):1403–1412. 10.1590/S0102-09352008000600015

[CR12] Eusebi V, Ichihara S, Vincent-Salomon A, Sneige N, Sapino A (2012) Exceptionally rare types and variants. In: Lakhani SR, Ellis IO, Schmitt SJ, Tan PH, van de Vijver MJ (eds) WHO classification of tumours of the breast, 4th edn. IARC Press, Lyon, pp 132–134

[CR13] Goldschmidt M, Peña L, Rasotto R, Zappulli V (2011) Classification and grading of canine mammary tumors. Vet Pathol 48(1):117–131. 10.1177/0300985810393258 21266722

[CR14] Hammond MEH, Hayes DF, Dowsett M, Allred DC, Hagerty KL, Badve S, Fitzgibbons PL, Francis G, Goldstein NS, Hayes M, Hicks DG, Lester S, Love R, Mangu PB, McShane L, Miller K, Osborne CK, Paik S, Perlmutter J, Rhodes A, Sasano H, Schwartz JN, Sweep FCG, Taube S, Torlakovic EE, Valenstein P, Viale G, Visscher D, Wheeler T, Williams RB, Wittliff JL, Wolff AC (2010) American Society of Clinical Oncology/College of American Pathologists guideline recommendations for immunohistochemical testing of estrogen and progesterone receptors in breast cancer. J Clin Oncol 28(16):2784–2795. 10.1200/JCO.2009.25.6529 PMC2881855 20404251

[CR15] Hughes K, Watson CJ (2018) The mammary microenvironment in mastitis in humans, dairy ruminants, rabbits and rodents: a one health focus. J Mammary Gland Biol Neoplasia 23:27–41. 10.1007/s10911-018-9395-1 PMC5978844 29705830

[CR16] Krings G, Chen YY, Sorensen PHB, Yang WT (2019) Secretory carcinoma. In: WHO Classification of Tu-mours Editorial Board (Eds) WHO Classification of Tumours - Breast Tumours. 5th edn. Lyon: International Agency for Research on Cancer, pp 146–149

[CR17] Künzel F, Grinninger P, Shibly S, Hassan J, Tichy A, Berghold P, Fuchs-Baumgartinger A (2015) Uterine disorders in 50 pet rabbits. J Am Anim Hosp Assoc 51(1):8–14. 10.5326/JAAHA-MS-5812 25415217

[CR18] Lavalle GE, Bertagnolli AC, Tavares WLF, Cassali GD (2009) Cox-2 expression in canine mammary carcinomas: correlation with angiogenesis and overall survival. Vet Pathol 46(6):1275–1280. 10.1354/vp.08-VP-0226-C-FL 19605908

[CR19] Li L, Wu N, Li F, Li L, Wei L, Liu J (2019) Clinicopathologic and molecular characteristics of 44 patients with pure secretory breast carcinoma. Cancer Biol Med 16(1):139–146. 10.20892/j.issn.2095-3941.2018.0035 PMC6528460 31119054

[CR20] Martín de las Mulas J, Millán Y, Dios R (2005) A prospective analysis of immunohistochemically determined estrogen receptor α and progesterone receptor expression and host and tumor factors as predictors of disease-free period in mammary tumors of the dog. Vet Pathol 42(2):200–212. 10.1354/vp.42-2-200 15753474

[CR21] McDivitt RW, Stewart FW (1966) Breast carcinoma in children. J Am Med Assoc 195(5):388–390. 10.1001/jama.195.5.388 4285563

[CR22] Millanta F, Calandrella M, Bari G, Niccolini M, Vannozzi I, Poli A (2005) Comparison of steroid receptor expression in normal, dysplastic, and neoplastic canine and feline mammary tissues. Res Vet Sci 79(3):225–232. 10.1016/j.rvsc.2005.02.002 16054892

[CR23] Min N, Zhu J, Liu M, Li X (2022) Advancement of secretory breast carcinoma: a narrative review. Ann Transl Med 10(21):1178. 10.21037/atm-22-2491 PMC9708487 36467350

[CR24] Nielsen TO, Leung SCY, Rimm DL, Dodson A, Acs B, Badve S, Denkert C, Ellis MJ, Fineberg S, Flowers M, Kreipe HH, Laenkholm AV, Pan H, Penault-Llorca FM, Polley MY, Salgado R, Smith IE, Sugie T, Bartlett JMS, McShane LM, Dowsett M, Hayes DF (2021) Assessment of Ki67 in breast cancer: updated recommendations from the international Ki67 in breast cancer working group. JNCI: J Natl Cancer Inst 113(7):808–819. 10.1093/jnci/djaa201 PMC8487652 33369635

[CR25] Osako T, Takeuchi K, Horii R, Iwase T, Akiyama F (2013) Secretory carcinoma of the breast and its histopathological mimics: value of markers for differential diagnosis. Histopathology 63(4):509–519. 10.1111/his.12172 23944930

[CR26] Peña L, Gama A, Goldschmidt MH, Abadie J, Benazzi C, Castagnaro M, Díez L, Gärtner F, Hellmén E, Kiupel M, Millán Y, Miller MA, Nguyen F, Poli A, Sarli G, Zappulli V, Martín de las Mulas J (2014) Canine mammary tumors: a review and consensus of standard guidelines on epithelial and myoepithelial phenotype markers, HER2, and hormone receptor assessment using immunohistochemistry. Vet Pathol 51(1):127–145. 10.1177/0300985813509388 24227007

[CR27] Ren H, Tong W, Ma J, Chen X, Huang H, Wei N, Liu Y, Yang M, Zhang L, Li H (2025) Secretory breast carcinoma: morphologic and molecular heterogeneity with indicators of aggressive potential in a cohort of 29 cases. J Pathol Clin Res 11(6):e70060. 10.1002/2056-4538.70060 PMC12590242 41195654

[CR28] Schöniger S, Horn LC, Schoon HA (2014) Tumors and tumor-like lesions in the mammary gland of 24 pet rabbits: a histomorphological and immunohistochemical characterization. Vet Pathol 51(3):569–580. 10.1177/0300985813497486 23892377

[CR29] Schöniger S, Degner S, Jasani B, Schoon HA (2019) A review on mammary tumors in rabbits: translation of pathology into medical care. Animals 9(10):762. 10.3390/ani9100762 PMC6826878 31581718

[CR30] Silva HC, Dos Reys MP, Cassali GD, Souza FR, Horta RS, Sena BV, Bindaco ALS, Pinto CJ, Souza TD, Flecher MC (2022) Secretory carcinoma of the canine mammary gland with nodal and bone metastases: Case report. Open Vet J 12(4):502. 10.5455/OVJ.2022.v12.i4.12 PMC9473362 36118721

[CR31] Tavassoli FA, Norris HJ (1980) Secretory carcinoma of the breast. Cancer 45:2404–2413. 10.1002/1097-0142(19800501)45:9<2404::aid-cncr2820450928>3.0.co;2-8 6445777

[CR32] Vaccaro E, Navas L, Ercolano M, Piegari G, Di Napoli E, Papparella S, Inverso D, Brunetti B, Paciello O, Russo V (2024) Immunohistochemical investigation of cyclooxygenase-2 expression in rabbit uterine adenocarcinoma and the potential use of COX-2 inhibitors in cancer therapy. Animals (Basel) 14(22):3169. 10.3390/ani14223169 PMC11590897 39595222

[CR33] Vasudev P, Onuma K (2011) Secretory breast carcinoma: unique, triple-negative carcinoma with a favorable prognosis and characteristic molecular expression. Arch Pathol Lab Med 135(12):1606–1610. 10.5858/arpa.2010-0351-RS 22129193

[CR34] Zappulli V, Peña L, Rasotto R, Goldschmidt MH, Gama A, Scruggs JL, Kiupel M (2019) Mammary tumors. In: Kiupel M (ed) Surgical pathology of tumors of domestic animals, vol Vol 2, 3rd ed. Davis-Thompson DVM Foundation, Gurnee, IL, USA, pp 49–53

[CR35] Zhao YY, Ge HJ, Yang WT, Shao ZM, Hao S (2024) Secretory breast carcinoma: clinicopathological features and prognosis of 52 patients. Breast Cancer Res Treat 203(3):543–551. 10.1007/s10549-023-07153-1 37897648

